# Acquired tonsillar herniation related to spontaneous intracranial hypotension: case reports

**DOI:** 10.3389/fneur.2024.1309718

**Published:** 2024-01-24

**Authors:** Lili Chen, Haijian Wu, Xingyue Hu, Guangyu Ying

**Affiliations:** ^1^Department of Neurology, Xiasha Campus, Sir Run Run Shaw Hospital, Zhejiang University School of Medicine, Hangzhou, Zhejiang, China; ^2^Department of Neurosurgery, Second Affiliated Hospital, Zhejiang University School of Medicine, Hangzhou, Zhejiang, China

**Keywords:** acquired tonsillar herniation, spontaneous intracranial hypotension, spontaneous spinal cerebrospinal fluid leak, neuroimaging, epidural blood patch

## Abstract

**Background:**

Acquired prolapse of the cerebellar tonsils in spontaneous intracranial hypotension (SIH) patients is rare. This study aims to evaluate neuroimaging changes of acquired prolapse of the cerebellar tonsils below the foramen magnum in SIH patients due to spontaneous spinal cerebrospinal fluid leakage, which was treated by targeted epidural blood patches (EBP).

**Methods:**

We retrospectively reviewed clinical and neuroimaging characteristics of 5 cases of SIH with acquired prolapse of the cerebellar tonsils that received targeted EBP in our institution from January 2013 to December 2016.

**Results:**

Of these SIH patients, all of them suffered from an orthostatic headache. Initial cranial MRI demonstrated descent of the cerebellar tonsils ≥5 mm. Intrathecal gadolinium-enhanced spinal MR myelography and/or spinal MR hydrography were performed to evaluate the level of spinal cerebrospinal fluid leakage. Symptoms were alleviated in all 5 patients after two (*n* = 4), or three (*n* = 1) targeted EBP during hospitalization. Follow-up cranial MRI revealed that the descent of cerebellar tonsils was reversed after EBP treatment.

**Conclusion:**

Acquired tonsillar herniation can occur in patients with SIH and spinal cerebrospinal fluid leakage. Symptoms of these patients may be resolved and radiologic findings may be reversed after EBP treatment.

## 1 Introduction

Spontaneous intracranial hypotension (SIH) is a rare but increasingly recognized neurological condition (1). Clinically, it is characterized by orthostatic headache and low cerebrospinal fluid (CSF) pressure associated with spontaneous non-traumatic spinal CSF leak (2). SIH patients may also present with other symptoms, such as photophobia, nausea, vomiting, cranial neuropathy, neck pain, radiculopathy, and even coma (3). Physical signs of neck stiffness, nystagmus, abducens nerve palsy, and bradycardia may be detected in SIH patients. There are several characteristic features of SIH in cranial magnetic resonance imaging (MRI), including subdural effusion, pachymeningeal enhancement, venous engorgement, pituitary enlargement, and brain sagging (4). The diagnosis of SIH is mainly based on clinical presentations and imaging findings.

Although SIH is typically manifested by orthostatic headaches, diagnosing SIH could be missed if not suspected (5). As reported (6, 7), some physicians may lack general familiarity with this condition, which has only received more widespread recognition within the past two decades. Conversely, Chiari malformation is a much more widely recognized cause of tonsil herniation on brain imaging. As a result, patients with tonsil herniation due to SIH may be misdiagnosed with Chiari malformation when tonsil herniation is present on their imaging while focusing solely on cerebellar tonsillar descent demonstrated on imaging. This misdiagnosis may result in delays in care and other potentially serious complications. Thus, we and other authors retrospectively reviewed these cases with an acquired tonsillar herniation due to spontaneous nontraumatic spinal CSF leak in SIH patients, which may help to prevent misdiagnosis and unnecessary surgery. The cerebellar tonsils of SIH patients may descend into the foramen magnum, which exhibits somewhat overlapping findings with Chiari malformation type I (CM-I) (6). It was reported that this subgroup of patients could be confused with CM-I and may even be incorrectly treated with suboccipital craniectomy for posterior fossa decompression (7, 8). However, other imaging features, such as diffuse and uniform pachymeningeal enhancement, disappearance of the CSF space, pons abnormally flattened against the clivus, and posterior fossa crowding, can help differentiate SIH-associated acquired tonsillar herniation from CM-I.

This article retrospectively reviewed the clinical data of 5 cases with an acquired tonsillar herniation due to spontaneous nontraumatic spinal CSF leak in SIH patients. Symptoms of these patients were resolved and radiologic findings were reversed after treatment with epidural blood patches (EBP).

## 2 Methods

From January 2013 to December 2016, the clinical and image data of 233 patients diagnosed with SIH in our institute were reviewed. The study was approved by the medical ethics committee of Sir Run Run Shaw Hospital, School of medicine, Zhejiang University. Informed consent for this study was obtained from all participants for treatment and publication. This survey follows Equator network guidelines (9). According to the diagnostic criteria of the 2nd edition International Classification of Headache Disorder (ICHD-2), a diagnosis of SIH was made. Patients who had previous head/spinal trauma, lumbar puncture, epidural anesthesia puncture, as well as other causes of CSF leak were excluded from this study. Opening pressure (OP) on lumbar puncture and cranial MRI were obtained for each patient. Also, spinal MRI coupled with intrathecal gadolinium-enhanced spinal MR myelography/MR hydrography was performed to evaluate the level of spinal CSF leakage. Brain and spine MRI, intrathecal gadolinium-enhanced spinal MR myelography or spinal MR hydrography was performed. Acquired tonsillar herniation was defined if cerebellar tonsils displaced ≥5 mm below the level of the foramen magnum. Targeted EBP was performed based on MR myelography/MR hydrography results. A follow-up cranial MRI was performed to assess imageological change, including the descent of cerebellar tonsils.

For the EBP treatment, all patients received EBP targeted to the assumed CSF leakage site after giving full informed consent. The EBP was performed under strict aseptic conditions. Patients were in a prone position and awake during the entire procedure. An 18G needle was inserted in the epidural space using a midline approach at the appropriate level (one or two vertebrae levels below CSF leakage) using the saline loss-of-resistance technique. Sterile autologous peripheral unclotted venous blood was slowly injected into the epidural space. The injection was stopped when the patient complained of radicular pain, numbness, or headache. The volume of injected blood was 10–15 mL depending on the condition. The patients were maintained in the prone position for 30 min after the procedure and then were placed in the supine position with strict bed rest for the next 48 h after EBP. A repeated EBP was considered at a minimum of a 1-week interval (10).

## 3 Results

### 3.1 Patients’ characteristics

Clinical and imaging features of these 5 SIH patients with acquired tonsillar herniation (5 females and 0 males) are summarized in Table 1, the mean age of them is 37.4 ± 4.7 years (range, 31–44). Of these SIH patients, 1 of them had a medical history of cervical spondylosis. All of them suffered from an orthostatic headache. In addition, 3 of them presented with benign paroxysmal positional vertigo, 3 of them presented with tinnitus, 1 of them presented with nausea and vomiting, and 1 of them presented with neck pain. A stiff neck was found by physical examination in one patient. The OP of patients was 0–55mmH2O, and spine MR hydrography/myelography indicated that CSF leakage was located at cervico-thoracic junction. All of them received EBP treatment at least twice. Symptoms were alleviated in all 5 patients after two (*n* = 4), or three (*n* = 1) targeted EBP during hospitalization. A follow-up cranial MRI was performed to assess imageological change. The last follow-up MRI was taken 57 days (range: 30–90 days) after EBP. It revealed that the descent of cerebellar tonsils was reversed after EBP treatment.

**Table 1:** Clinical characteristics of 5 cases of SIH with acquired tonsillar herniation treated by targeted epidural blood patches.

Case	Age (ys), sex	Past medical history	Symptom, sign	OP (mmH2O)	Descent of the cerebellar tonsils on MRI (mm)	Locations of CSF leaks		Treatment	Outcome
						Spine MR hydrography	Spine MR myelography		
1	41, female	None	Orthostatic headache, benign paroxysmal positional vertigo, tinnitus	0	5	Cervico-thoracic junction	Cervico-thoracic junction	Targeted EBP, twice	Resolved
2	34, female	Cervical spondylosis	Orthostatic headache, tinnitus	25	6	C5–T7	C5–T2	Targeted EBP, three times	Resolved
3	31, female	None	Orthostatic headache, nausea and vomiting, tinnitus	42	6	Cervico-thoracic junction	Cervico-thoracic junction	Targeted EBP, twice	Resolved
4	37, female	None	Orthostatic headache, benign paroxysmal positional vertigo, tinnitus	55	5	Cervico-thoracic junction	Cervico-thoracic junction	Targeted EBP, twice	Resolved
5	44, female	None	Orthostatic headache, benign paroxysmal positional vertigo, cervicodynia	0	5	Cervico-thoracic junction	Unsuccess	Targeted EBP, twice	Resolved

CSF, cerebrospinal fluid; EBP, epidural blood patches; MR, magnetic resonance; OP, opening pressure; SIH, spontaneous intracranial hypotension; ys, years.

### 3.2 Illustrative case

**Figure 1:**
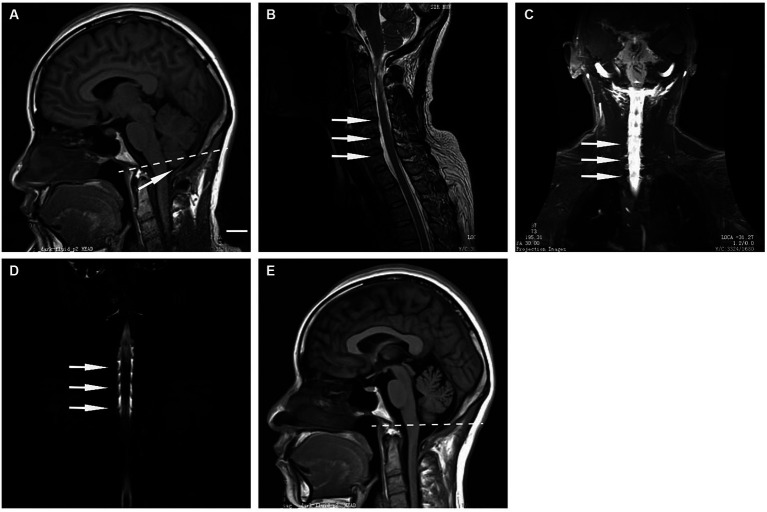
**(A)** Sagittal T1-weighted MRI performed at 1 month after the onset of headaches showing 5 mm prolapse of the cerebellar tonsils (arrow) into the foramen magnum (dashed line). **(B)** Sagittal T2-weighted MRI showed a crowded spinal canal. **(C)** Intrathecal gadolinium-enhanced MR myelography showed CSF leakage in the cervico-thoracic junction. **(D)** MR myelography indicated that the CSF leakage site was located in the cervico-thoracic junction. **(E)** Sagittal T1-weighted MRI performed at 2 months after targeted EBP intervention showed that re-ascent of the cerebellar tonsils above the foramen magnum (dashed line). Scale bar: 1 cm.

Case 1 was a 41-year-old female suffered from orthostatic headache, benign paroxysmal positional vertigo, and tinnitus for 1 month. She had no past medical history. Physical examination did not reveal any other abnormal signs. Lumbar puncture for pressure testing revealed the OP of this patient is 0mmH2O. Initial cranial MRI showed the descent of cerebellar tonsils below the foramen magnum is 5 mm (Figure 1A). Sagittal image of T2-weighted MRI showed the spinal canal is crowded (Figure 1B). Intrathecal gadolinium-enhanced MR myelography demonstrated that spinal CSF leakages (Figure 1C) were observed in the cervico-thoracic junction. Also, spinal MR myelography (Figure 1D) demonstrated that CSF leakage sites were located in the cervico-thoracic junction. The patient was treated with targeted EBP twice. After treatment, the patient’s symptom was resolved. MRI showed that the cerebellar tonsil was located in the anatomical position above the foramen magnum 2 months later (Figure 1E).

## 4 Discussion

In this study, we reviewed the clinical data of 5 SIH cases with a Chiari-like tonsillar herniation secondary to spontaneous nontraumatic spinal CSF leak. Following targeted EBP, symptoms were resolved and radiologic findings were revised. Considering SIH patients may present with several clinical and neuroradiological findings similar to those observed in CM-I cases, it is important to use neuroradiological and myelographic techniques for differential diagnosis and ultimately promote the most appropriate treatment for each patient.

Typically, SIH results from spontaneous CSF leak, most of which occur in the spine, particularly the level of cervicothoracic junction and thoracic spine (5). Even though the exact cause of spontaneous spinal CSF leak remains unclarified, factors including weakness of the meningeal sac and trivial trauma are considered (11). Because that CSF provides protective buoyancy to intracranial structures, spontaneous CSF leakage and loss of CSF volume can cause brain sagging. This may stretch or distort pain-sensitive anchoring structures of the brain, which therefore resulting in orthostatic headaches (12). Of note, orthostatic headache is a characteristic symptom for SIH patients. It occurs when standing up and is relieved with recumbency. Also, patients with SIH can be manifested with other symptoms, including nausea, vomiting, dizziness, neck pain or stiffness, photophobia, diplopia, visual blurring, change in hearing, and back pain (13). Indeed, there is considerable variance in clinical manifestation and imaging findings. Typically, CSF pressure of SIH patients is very low, sometimes it is unmeasurable, but occasionally is even consistently within normal limits. Fortunately, more and more cases of SIH than before have been diagnosed with the help of MRI scan (14). Cranial MRI of SIH patients may reveal the following abnormalities, including subdural fluid collection, diffuse pachymeningeal enhancement, decreased ventricular size, enlarged pituitary, as well as engorged cerebral venous sinuses. Of important, the brain sagging of this disease can be manifested by descent of the cerebellar tonsils, and sometimes even similar to CM-I, a congenital condition recognized by herniation of the cerebellar tonsils into the foramen magnum (6). Atkinson et al., reported that in total of 35 SIH patients, 7 of them showed tonsillar descent to and even below the C1 posterior arch (15). Puget et al., reported a Chiari-like tonsillar herniation in a 12-year-old SIH patient with Marfan syndrome (7). This patient suffered from a postural headache and accepted posterior fossa decompression, however, clinical symptoms became worse after surgery (7). Later, MR myelography was performed and indicated a relevant CSF leak at S1. The pain was completely resolved and the cerebellar tonsil displacement was reversed after two sessions of EBP. Thus, due to its overlapping findings with CM-I, diagnosis of SIH should be somewhat cautious (16). A Chiari-like cerebellar tonsillar descent does not ensure the accuracy of CM-I diagnosis (8). More clinical and imaging techniques to evaluate the CSF leaks are anticipated, which could promote accurate diagnosis and treatment. In order to clarify the location of CSF leak, computed tomography (CT) and/or MR myelography are recommended. CT myelography is a preferred technique for localizing high-flow spinal CSF leaks, while MR myelography with intrathecal gadolinium is relatively sensitive for intermittent or low-flow leaks (17).

A spectrum of treatment methods has been implemented for SIH patients, involving bed rest, caffeine or theophylline, fluid supplementation, EBP, or surgical repairs of the leak (1). Fortunately, many patients could recover with increased fluid intake and strict bed rest. If an initial trial of conservative management has failed, EBP is advocated. It could be delivered blindly into the lumbar region or targeted to the specific site of CSF leak (18). Of note, controversy still exists over the optimal strategy for EBP delivery in the treatment of SIH. Several studies indicated that targeted EBP is likely to be more effective than blindly delivered EBP in treating this disease (13, 19). For instance, in a retrospective non-randomized controlled study, Cho et al. found that 27 of 31 SIH patients (87.1%) exhibited clinical improvement after receiving a targeted EBP during first administration (20). In contrast, 13 of 25 SIH patients (52%) achieved complete recovery after receiving a blind EBP via a lumbar or upper thoracic epidural route (20). Also, it reported that lower proportions of SIH patients required repeat EBP after initially targeted EBP treatment when compared with those initially treated with blind EBP (21 and 61% respectively) (21). Additionally, Feltracco et al. demonstrated that a thoracic-targeted EBP treatment is favorable in improving clinical outcomes when compared to those received a lumbar EBP (22). Both quality of headache relief and low incidence of recurrence was observed in patients with thoracic-targeted EBP during long-term follow-up (22). In our case series, all 5 SIH patients received at least 2 targeted EBP and gained a favorable outcome. With accurate localization of spinal CSF leak, repeat and targeted EBP could be safe and effective (23). On the other hand, when compared patients who initially received two-site blind EBP at the cervicothoracic (C7/T1) and thoracolumbar junctions (T12/L1), Ahn et al. found that no significant outcome difference was detected after receiving targeted approaches (23). Of note, targeted EBP maybe have a higher risk of complications, such as neck stiffness, chemical meningitis, and compression of spinal cord and nerve roots (20, 21). Thus, no consensus has yet been reached and prospective, randomized and controlled clinical trials comparing targeted with blind EBP are anticipated.

In many cases, EBP may seal the leak successfully corresponding to the level of the leak, resulting in a favorable outcome (2, 12, 24). But the large leak may not be respond to this minimally invasive approach. More invasive surgical approaches could only be tried in well-chosen cases (25, 26). In our cases, acquired tonsillar herniation is linked to spontaneous nontraumatic spinal CSF leak, symptoms and radiologic findings of the herniation of cellular tonsillar are reversed after targeted EBP treatment. Thus, suitable therapeutic strategies should be tailored to treat acquired tonsillar herniation in SIH patients due to spontaneous nontraumatic spinal CSF leaks. Of note, the study lacks a control group and does not compare the procedure’s benefits to other treatments. Also, there is a lack of objective scoring for symptom changes before and after EBP treatment, such as VAS scores, which are anticipated to be included in future studies.

## 5 Conclusion

Acquired tonsillar herniation can occur in SIH patients. Appropriate therapeutic strategies for this disease can revert the cerebellar tonsil displacement and improve patients’ outcomes.

## Data availability statement

The original contributions presented in the study are included in the article/supplementary material, further inquiries can be directed to the corresponding author.

## Ethics statement

The study was approved by the medical ethics committee of Sir Run Run Shaw Hospital, School of medicine, Zhejiang University. Informed consent was obtained from all individual participants for treatment and publication.

## Author contributions

LC: Data curation, Formal analysis, Methodology, Writing – original draft. HW: Data curation, Formal analysis, Methodology, Writing – original draft. XH: Writing – review & editing. GY: Conceptualization, Supervision, Writing – review & editing.
